# Increased Safety Behavior and COVID-19-Related Fear in Adults with Cystic Fibrosis during the Pandemic

**DOI:** 10.3390/healthcare10050858

**Published:** 2022-05-06

**Authors:** Anke-Verena Benecke, Kira Leandra Schmidt, Hannah Dinse, Adam Schweda, Lisa Jahre, Madeleine Fink, Benjamin Weismüller, Nora Dörrie, Matthias Welsner, Eva-Maria Skoda, Alexander Bäuerle, Venja Musche, Martin Teufel

**Affiliations:** 1 Clinic for Psychosomatic Medicine and Psychotherapy, LVR-University Hospital Essen, University of Duisburg-Essen, 45147 Essen, Germany; anke-verena.benecke@lvr.de (A.-V.B.); hannah.dinse@uni-due.de (H.D.); adam.schweda@uni-due.de (A.S.); lisa.jahre@lvr.de (L.J.); madeleine.fink@uni-due.de (M.F.); benjamin.weismueller@uni-due.de (B.W.); nora.doerrie@lvr.de (N.D.); eva-maria.skoda@uni-due.de (E.-M.S.); alexander.baeuerle@uni-due.de (A.B.); venja.musche@uni-due.de (V.M.); martin.teufel@lvr.de (M.T.); 2 Center for Translational Neuro- and Behavioral Sciences (C-TNBS), University of Duisburg-Essen, 45147 Essen, Germany; 3 Department of Pulmonary Medicine, University Hospital Essen—Ruhrlandklinik, Adult Cystic Fibrosis Center, University of Duisburg-Essen, 45147 Essen, Germany; matthias.welsner@rlk.uk-essen.de

**Keywords:** cystic fibrosis, COVID-19, SARS-CoV-2, safety behavior, coping, mental health burden

## Abstract

People with cystic fibrosis (pwCF) face great challenges during the ongoing COVID-19 pandemic. Recent research found equal levels of distress in pwCF and healthy controls (HC). The current study aimed to investigate the mental health burden and safety behavior in pwCF. Sixty-nine adult pwCF and sixty-nine propensity-score-matched HC participated in this study. Participants completed an anonymous online questionnaire assessing distress, generalized anxiety, depressive symptoms, COVID-19-related variables, self-reported adherent safety behavior (ASB), and dysfunctional safety behavior (DSB). PwCF showed equal amounts of distress (*W* = 2481.0, *p* = 0.669), depressive symptoms (*W* = 2632.5, *p* = 0.268), and generalized anxiety symptoms (*W* = 2515.5, *p* = 0.565) compared to the HC. COVID-19-related fear (*W* = 1872.0, *p* = 0.028), ASB (*W* = 1630.0, *p* = 0.001), and DSB (*W* = 1498.5, *p* < 0.001) were significantly elevated in pwCF. The pwCF estimated that the probability of suffering from symptoms (*W* = 954.5, *p* < 0.001), experiencing a severe course (*W* = 806.5, *p* < 0.001), or dying (*W* = 1079.0, *p* < 0.001) from COVID-19 is significantly higher than that of the HC. ASB was associated with a CF diagnosis, COVID-19-related fear, and a subjective level of information (*R^2^* = 0.414, *F*(13, 124) = 6.936, *p* ≤ 0.001). DSB was associated with a diagnosis of CF and COVID-19-related fear (*R^2^* = 0.196, *F*(13, 124) = 3.169, *p* ≤ 0.001). The data suggest that pwCF show functional and adequate behaviors towards the risk caused by the pandemic. Therefore, functional coping behaviors may provide advantages in addressing pandemic challenges.

## 1. Introduction

Based on the ongoing global spread of SARS-CoV-2 (COVID-19), daily life is affected by protective regulations and contact bans within different periods of lockdown. Clinical symptoms of the COVID-19 infection range from asymptomatic or mild symptoms, to severe illness, from dyspnea and pneumonia to critical respiratory failure, septic shock, and/or multi-organ failure [1]. Due to the fear of contracting COVID-19, mental health burdens increased in the German population [2]. In particular, distress, generalized anxiety, and depressive symptoms, as well as COVID-19-related fear, were prevalent in the German public [2]. Additionally, risk groups for developing a severe or fatal course in cases of COVID-19 infections have been defined [3,4], including individuals with different chronic lung diseases, such as people with cystic fibrosis (pwCF). Cystic fibrosis (CF) is a life-limiting, monogenetic, and autosomal-recessive disease affecting up to 8000 people in Germany [5,6], which leads to irreversible damage, especially of lung parenchyma [7]. Accordingly, infectious diseases of the respiratory tract, such as COVID-19, pose a major risk for pwCF, as they can further impair the function of the already affected lung [8].

Since the beginning of the pandemic, people with risk factors have been advised to avoid contact and to implement special hygiene measures. Therefore, the German Cystic Fibrosis Association [9] published corresponding behavioral and hygiene recommendations for those affected. Recent research regarding the mental health burden during the COVID-19 pandemic indicates that of all individuals with high-risk diseases, those with chronic respiratory diseases show the highest anxiety levels [10]. Even without the COVID-19 pandemic, pwCF face unique challenges in maintaining good physical health, such as good physical conditions, taking medications regularly, exercising, attending follow-up examinations, and adhering to specific hygiene measures. Due to COVID-19-related restrictions in public life, such as the closure of gyms and public sports fields, adherence to physical activity is significantly reduced, even though it represents an essential component for improving life expectancies of pwCF [11].

Although pwCF face particular challenges during the ongoing pandemic, a recent study reported no significant difference in psychological distress between pwCF and healthy controls (HC) [12]. The authors hypothesized that the lifelong experiences of pwCF, in coping with the demands of their disease, might explain this finding. Since research is lacking, the current study examined not only the mental health burden of pwCF during the COVID-19 pandemic compared to HC, but also COVID-19-related fear, risk perceptions, and the subjective level of information regarding COVID-19 regulations. Moreover, self-reported behavioral changes, such as adherent safety behavior (ASB; adherence to hygiene and distance rules, i.e., increased hand washing) and dysfunctional safety behavior (DSB, i.e., increasing selfish behavior) were investigated.

The aim of the current study was to investigate the mental health burden of pwCF during the COVID-19 pandemic as well as COVID-19-related fear, risk perception, and behavioral changes, i.e., ASB and DSB, and compare these to a group of HC. In accordance with Ciprandi et al. [12] and previous literature [13,14], the current study assumes that pwCF will exhibit equal levels of distress, anxiety, and depressive symptoms compared to HC during the COVID-19 pandemic. However, pwCF are expected to have increased subjective risk perceptions regarding COVID-19 infections, suffering from COVID-19-related symptoms, severe progression, and dying from COVID-19 due to their underlying disease. To follow up on the findings of Ciprandi et al., the current study expects pwCF to show significantly more ASB than the general population, due to their lifelong experience with hygiene measures and precautions, and assumes an elevated DSB in accordance with increased anxiety levels [15]. Additionally, COVID-19-related fear and subjective levels of information were expected to be associated with either ASB or DSB.

## 2. Materials and Methods

### 2.1. Participants and Procedures

A cross-sectional study was conducted, in which 69 pwCF and 69 propensity-score-matched HC in Germany participated during the first lockdown from 1 April to 26 May 2020. The pwCF were recruited via an e-mail list distributed by a CF self-help group. The control group was collected from a large sample of 14,190 participants from an online survey on the topic of COVID-19, which has been reported in previous research [2,13,14,16].

Requirements for participation were age of majority (≥18 years), a good command of the German language, and internet access. For the sample with pwCF, a CF diagnosis was also required. All participants provided electronic informed consent. The Ethics Committee of the University Hospital Essen (20-9307-BO) approved the study.

### 2.2. Propensity Score Matching

Propensity Score Matching (PSM) [17] was used to account for the differences between the two samples and to condition on the large set of covariates [18,19]. Due to the influence of potentially confounding covariates (e.g., via sociodemographic characteristics), PSM aimed to remove bias in order to estimate the effect of a given variable [18]. Cases were matched based on age, gender, marital status, educational level, previous mental illness, and size of residence. Logistic regression was used to estimate the propensity score.

### 2.3. Assessment Instruments

All participants completed an anonymous online questionnaire including sociodemographic and medical data, validated mental health assessment instruments, and self-generated items on COVID-19-related perception and behavior. Sociodemographic and medical data included gender, age, marital status, educational level, and city size for all participants. Both groups were asked about previous mental disorders. PwCF responded to items regarding the time since their diagnosis of CF and the severity of their symptoms. Distress was investigated with the German version of the Distress Thermometer, on a scale ranging from 0 = “no distress” to 10 = “extreme distress” (scores ≥ 5 indicates elevated distress) [20]. The Generalized Anxiety Disorder Scale-7 (GAD-7) was used to measure general anxiety symptoms with seven items on a 4-point Likert-scale (0 = “not at all” to 3 = “nearly every day”) with a Cronbach’s α of 0.89 [21]. Scores of ≥5, ≥10, and ≥15 denoted mild, moderate, and severe anxiety symptoms, respectively. The Patient Health Questionnaire-2 (PHQ-2) was used to assess depressive symptoms in the past two weeks on a 4-point Likert-scale (0 = “not at all” to 3 = “nearly every day”) with a Cronbach’s α of 0.84 [22]. Scores of 3 and above were considered thresholds for major depression symptoms. Three self-generated items were used to assess the subjective level of information regarding COVID-19 (“I feel informed about COVID-19 (corona virus)”, “I feel informed about measures to avoid an infection with COVID-19”, and “I understand the guidance from health authorities regarding COVID-19”) [2,13]. Responses were given on a 7-point Likert-scale (1 = “strongly disagree” to 7 = “strongly agree”). Its internal consistency was a Cronbach’s α of 0.71. Adherent safety behavior (ASB; adherence to hygiene and distance rules, i.e., increased hand washing, avoidance of public places and public transportation, change of travel plans) and dysfunctional safety behavior (DSB, i.e., buying large quantities of hygiene products and staple foods, increasing selfish behavior) were assessed using four items from the ASB sub-scale and four items from the DSB sub-scale. The items were answered on a 7-point Likert-scale (1 = “strongly disagree” to 7 = “strongly agree”) with a Cronbach’s α of 0.82 for ASB and 0.72 for DSB [13]. In addition, participants reported their subjective risk perception of becoming infected with COVID-19, suffering from COVID-19-related symptoms, having a severe course, and dying from COVID-19, as percentages (0–100%).

### 2.4. Statistical Analyses

Statistical analyses were performed using SPSS Statistics version 26 (IBM, New York, NY, USA). The R package *MatchIt* was used for Propensity Score Matching [23]. Figures were created using SPSS Statistics version 26 (IBM, New York, NY, USA) and Adobe InDesign CC (Adobe Inc., Dublin, Ireland). Since Kolmogorov–Smirnov Tests revealed that the normality of the data was violated, Wilcoxon-signed-rank tests were performed to compare pwCF and matched HC. Effect sizes were defined using Cliff’s *δ* [24]. Here, effect sizes were interpreted according to Romano and colleagues (2006), where *d* = 0.147 represented a small effect, *d* = 0.33 represented a medium-sized effect, and *d* = 0.474 represented a large effect [25]. For all analyses, the significance levels were set at *α* = 0.05 (two-sided tests). Moreover, a subsequent regression analysis was calculated for both pwCF and healthy controls (*n* = 138) using robust Huber–White standard errors with diagnoses, COVID-19-related fear, and subjective level of information predicting either ASB or DSB.

## 3. Results

### 3.1. Participant Characteristics

Initially, 133 pwCF participated in the survey, 78 of which completed the questionnaire. Out of these, 9 individuals were excluded because they did not meet the required inclusion criteria (*n* = 69). The proportion of evaluable questionnaires was 58.65%. Table 1 displays sociodemographic data of pwCF and HC.

### 3.2. Emotion and Behavior

There were no significant differences between pwCF and HC in terms of general distress during the pandemic (Table 2), i.e., distress and generalized anxiety and depressive symptoms. However, both groups presented mild anxiety symptoms on average (CF: *M* = 6.0, *SD* = 4.00; HC: *M* = 7.26, *SD* = 5.86). COVID-19-related fear was significantly elevated in pwCF compared to HC (*W* = 1872.0, *p* = 0.028, *d* = −0.214). PwCF showed significantly more ASB (*W* = 1630.00, *p* = 0.001, *d* = −0.315), as well as DSB (*W* = 1498.5, *p* < 0.001, *d* = −0.371), than HC. Significantly associated with ASB were diagnoses of CF, COVID-19-related fear, subjective levels of information, and age being between 65 and 74 years (*R^2^* = 0.414, *F*(13, 124)= 6.936, *p* ≤ 0.001). DSB was significantly associated with a diagnosis of CF and COVID-19-related fear (*R^2^* = 0.196, *F*(13, 124) = 3.169, *p* ≤ 0.001) (see Supplements Tables S1 and S2). Since the sample size was small, we conducted a post hoc power analysis using G*Power [26,27], which revealed a power of 0.97.

### 3.3. Subjective Risk Perception

**Figure 1:**
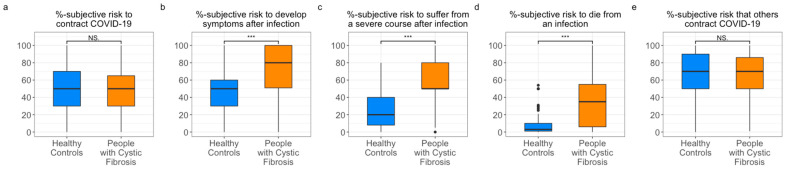
Distribution of risk perception, in percent (%), in people with cystic fibrosis (pwCF) compared to healthy controls (HC), regarding (**a**) risk of infection with COVID-19, (**b**) presenting symptoms of COVID-19, (**c**) having a severe course of COVID-19, (**d**) risk of dying of COVID-19, and (**e**) risk that others contract COVID-19. For descriptive statistics see Table 2.

PwCF rated the risk of becoming infected with COVID-19 to be the same as the HC (*W* = 2546.0, *p* = 0.480, *d* = 0.07; Table 2). The probability of suffering from a symptomatic course (*W* = 954.5, *p* < 0.001, *d* = −0.599), a severe course (*W* = 806.5, *p* < 0.001, *d* = −0.661), or death (*W* = 1079.0, *p* < 0.001, *d* = −0.547) in the event of infection with COVID-19 was significantly higher in pwCF (Figure 1).

## 4. Discussion

The current study investigated the mental health burden of people with cystic fibrosis (pwCF) during the ongoing COVID-19 pandemic, as well as COVID-19-related fear, risk perception, and behavioral changes, in comparison to propensity-score-matched healthy controls (HC). With 69 evaluable questionnaires, close to 1% of pwCF in Germany participated in the current study [6]. The data show comparable manifestations of distress, anxiety, and depressive symptoms in pwCF and HC. However, pwCF showed more COVID-19-related fear and estimated their risk of developing symptoms, a severe course, or death in the event of a COVID-19 infection, significantly higher than the HC. Moreover, pwCF showed significantly more adherent safety behavior (ASB) and dysfunctional safety behavior (DSB) compared to HC. CF diagnoses, COVID-19-related fear, subjective levels of information, and ages between 65 and 74 years were significantly associated with ASB. DSB was associated with CF diagnoses and COVID-19-related fear.

Since recent studies suggest that the mental health burdens of individuals belonging to high-risk groups is comparable to HC during the COVID-19 pandemic [12,13,14], the current study expected equal levels of distress, anxiety, and depressive symptoms in pwCF compared to HC. Even though pwCF are faced with a higher risk regarding COVID-19 infections [3,4], the current data showed a comparable mental health burden of pwCF and HC. These findings are consistent with Ciprandi et al. (2021) [12]. This suggests that the ongoing pandemic does not lead to an increased psychological burden in pwCF. Nonetheless, both groups exhibited mild anxiety symptoms, reflecting the strains the pandemic places on everyone. This is in line with previous literature showing that psychological distress is elevated during the current pandemic [2].

In this study, pwCF reported a significantly higher risk perception than HC. While they perceived the likelihood of contracting COVID-19 to be similar to HC, they reported an increased risk of suffering from symptoms, having a severe course, or dying in case of an infection with COVID-19, which are realistic estimations in terms of their risk profiles [3,4]. Therefore, it is not surprising that pwCF displayed higher risk perceptions compared to HC, who face a, realistically, lower risk due to the absence of pre-existing diseases.

Moreover, increased COVID-19-related fear was found in the present study compared with HC, which could be a consequence of increased risk perception. Due to the fact that an infection with COVID-19 poses a realistic threat to pwCF [3,4], the elevated COVID-19-related fear could be interpreted as an adequate functional response. Recent research proposes to differentiate between generalized anxiety and COVID-19-related fear [28,29,30], whereby generalized anxiety is often referred to as panic leading to sensory restriction and avoidance behaviors, whereas ‘fear’ represents the concern regarding an object or situation leading to activism. Increased COVID-19-related fear could also be considered a warning function and a need for security, which is reflected in elevated safety behaviors (i.e., ASB and DSB). Therefore, it is not surprising that the current study found increased ASB and DSB in pwCF compared to HC. PwCF showed significantly more ASB, such as increased hand washing and avoidance of public places, as well as DSB, such as hoarding of hygiene products and staple foods. All of these actions and behaviors are aimed at protecting against a COVID-19 infection and can therefore be considered functional coping behaviors. Due to their disease, pwCF could see themselves in situations where they have to take precautions in order to maintain good physical conditions, even before the pandemic. These lifelong experiences in managing their disease might explain the fact that pwCF do not display elevated mental health burdens when confronted with the current pandemic compared with HC. Although the pwCF reported increased COVID-19 fear and risk perception, this functional safety behavior might not represent a new challenge for these patients. In contrast, HC have to face new challenges, such as increased hygiene measures and social distancing, in order to deal with the pandemic, which could result in increased psychological burdens, as noted in previous literature [2,31]. The results of the subsequent regression analysis of the present study support these findings by showing that ASB and DSB were associated with the presence of a CF diagnosis and COVID-19-related fear. Thus, the present study was able to extend the findings from Ciprandi et al. (2021) [12]. The data support the hypothesis that the reason pwCF do not show an increased mental health burden compared to HC, despite facing particular challenges during the pandemic, may be due to their lifelong experiences of managing their disease.

Interestingly, in previous literature, similar results were found in individuals from risk groups, i.e., patients with diabetes or cancer [13,14]. Here, compared to HC, patients did not show increased levels of distress and general anxiety, but did show increased ASB and DSB.

With regard to the course of mental stress during the COVID-19 pandemic, longitudinal studies showed that pandemic lockdowns have small but significant effects on mental health, such as increased anxiety and depression [32]. However, even though mental health symptoms increased at the beginning of the pandemic, the psychological burden decreased after the lockdown [33] and was comparable to pre-pandemic levels by mid-2020 [34]. These results suggest that people might get used to the measures that need to be conducted during pandemic circumstances. Since we assume that the coping behavior of pwCF might constitute a protective role for mental health, and those patients are already used to conducting special hygiene measures in order to maintain their physical health, this might explain why pwCF show comparable mental health burdens, similar to HC, even though they experience higher risks of getting infected by COVID-19. The results of the current study indicate that the ongoing COVID-19 pandemic is affecting everyone’s lives and leads to an increased mental health burden, regardless of pre-existing medical conditions [2]. However, functional coping behavior might pose a protective role for the mental health of pwCF and could provide an advantage in addressing the unique challenges caused by the pandemic. If we assume that these coping behaviors lie at the core of dealing with the pandemic, it is important to implement psychological support strategies, such as online therapies and e-mental health training, in order to support coping strategies in the general population [35].

### Limitations

The current study displays a few limitations. First, the present study includes a small sample size of *n* = 138. However, post hoc power analysis reveals an adequate power of 0.97. Additionally, there was a gender imbalance in the present study, since mainly female individuals participated in the survey. A previous study showed higher levels of anxiety, depression, and somatization, but also more coping behavior in women than in men as reaction to COVID-19 [36]. This might also have influenced the psychological reactions of the present sample. Moreover, since only adult patients were included, it is not possible to relate the results to adolescents or children. Further, due to self-reported data, no validation of the CF diagnosis is possible. In addition, it was mainly pwCF who are in relatively good physical and mental health condition who participated in the survey. In addition, no information regarding confirmed COVID-19 infections of our sample is available. Moreover, selection bias might have resulted from the recruitment of pwCF via a support group e-mail list. This might have mainly included pwCF who were especially in need of support or were open-minded regarding CF-related safety measures and topics. Because the data were collected in a cross-sectional study, no causal conclusions can be drawn. The safety behavior scales and the scale’s subjective level of information regarding COVID-19 have not been previously validated. However, post hoc validation of the established scale showed high internal consistency [2,37]. Nevertheless, our study provides an important impetus for a larger project that aims to reduce bias.

## 5. Conclusions

In conclusion, the current COVID-19 pandemic poses a significant threat to the general population and especially to people belonging to at-risk groups, such as pwCF. PwCF show similar levels of distress and generalized anxiety and depressive symptoms as HC. However, they displayed elevated COVID-19-related fear and reported increased risk perceptions and safety behaviors. The results indicate that pwCF adequately assess the threat posed by the current pandemic and respond in a functional manner. Nonetheless, psychological care structures could be implemented in order to meet the needs of this patient group and support their long-term mental health.

## Figures and Tables

**Table 1:** Sociodemographic data.

	People with CF		Healthy Controls		
	*n*	%	*n*	%	*p*
**Sex**					1
Female	59	85.5	60	87.0	
Male	10	14.5	9	13.0	
**Age**					0.984
18–24 years	11	15.9	9	13.0	
25–34 years	22	31.9	25	36.2	
35–44 years	20	29.0	21	30.4	
45–54 years	11	15.9	9	13.0	
55–64 years	4	5.8	4	5.8	
65–74 years	1	1.4	1	1.4	
**Marital status**					0.935
Single	20	29.0	20	29.0	
Married	25	36.2	26	37.7	
In a relationship	17	24.6	17	24.6	
Divorced/separated	4	5.8	2	2.9	
Others	3	4.3	4	5.8	
**Educational level**					0.959
University education	21	30.4	22	31.9	
Higher education entrance qualification	22	31.9	24	34.8	
Intermediate secondary education	18	26.1	17	24.6	
Lower secondary education	3	4.3	3	4.3	
Others	5	7.2	3	4.3	
**City size**					0.995
100,000 residents	18	26.1	18	26.1	
20,000 residents	20	29.0	20	29.0	
5000 residents	10	14.5	11	15.9	
<5000 residents	21	30.4	20	29.0	
**Mental disorders**					1
yes	14	20.3	13	18.8	
no	55	79.7	56	81.2	
**Total**	69	100	69	100	

**Table 2:** Comparisons between people with CF and healthy controls.

	People with CF (*n* = 69)		Healthy Controls (*n* = 69)				
	*M (SD)*	*Median (IQR)*	*M (SD)*	*Median (IQR)*	*W*	*p*	*Cliff’s* *δ*
Distress	5.38 (2.79)	6 (5)	5.55 (2.89)	6 (5)	2481.0	0.669	0.042
Generalized anxiety symptoms	6.0 (4.00)	5 (5)	7.26 (5.86)	5 (6)	2515.5	0.565	0.057
Depressive symptoms	1.52 (1.39)	2 (2)	2.03 (1.95)	2 (3)	2632.5	0.268	0.106
COVID-19-related fear	4.91 (1.82)	5 (2)	4.22 (1.90)	5 (3)	1872.0	0.028 *	−0.214
Subjective level of information	6.01 (0.76)	6 (1.33)	5.80 (1.0)	6 (1)	2091.5	0.213	−0.121
ASB	6.28 (0.97)	6.75 (1)	5.48 (1.58)	6 (2)	1630.0	0.001 **	−0.315
DSB	3.46 (1.48)	3.67 (2.33)	2.52 (1.5)	2 (1.67)	1498.5	<0.001 **	−0.371
**Risk perception**							
Infection with COVID-19	47.94 (24.88)	50 (35)	51.09 (27.57)	50 (40)	2546.0	0.48	0.07
Suffering from symptoms	74.59 (25.45)	80 (49)	46.93 (22.55)	50 (30)	954.5	<0.001 **	−0.599
Having a severe course	58.9 (27.77)	50 (30)	24.06 (20.42)	20 (32)	806.5	<0.001 **	−0.661
Dying of COVID-19	36.33 (31.07)	35 (49)	9.84 (14.24)	3 (8)	1079.0	<0.001 **	−0.547
Risk that others contract COVID-19	63.43 (28.56)	70 (36)	63.68 (31.22)	70 (40)	2440.5	0.799	0.025

Note. Mean parameter values and median for each of the analyses are shown for people with CF (*n* = 69) and healthy controls (*n* = 69), as well as the results of Wilcoxon-signed-rank tests (assuming unequal variance). ASB = adherent safety behavior, DSB = dysfunctional safety behavior. ** *p* ≤ 0.01 * *p* ≤ 0.05.

## Data Availability

The raw data supporting the conclusions of this article will be made available by the corresponding author, on reasonable request.

## Supplementary Materials

The following supporting information can be downloaded at: https://www.mdpi.com/article/10.3390/healthcare10050858/s1, Table S1: Regression coefficients predicting ASB; Table S2: Regression Coefficients Predicting DSB.
