# Supplementary material for: Increased Safety Behavior and COVID-19-Related Fear in Adults with Cystic Fibrosis during the Pandemic
Source: Healthcare (Basel). 2022 May 6;10(5):858. doi: 10.3390/healthcare10050858 (PMC9142094; doi:10.3390/healthcare10050858)
Supplement: Supplementary file 1 [file healthcare-10-00858-s001.zip › healthcare-1682072-supplementary.pdf]

Table S1. Regression coefficients predicting ASB.

	Estimates	*CI*	*p*
Intercept	2.42	.97 – 3.88	.001**
Diagnosis of CF	0.51	.12 – .90	.011*
COVID-19-related fear	0.32	.19 – .46	<.001**
Subjective level of information	0.32	.09 – .54	.006**
Age: 25-34, ref: 18-24	-.06	-.58 – .46	.829
Age: 35-44, ref: 18-24	-.3	-.84 – .23	.267
Age: 45-54, ref: 18-24	-.2	-.70 – .30	.428
Age: 55-64, ref: 18-24	-.82	-2.10 – .46	.207
Age: 65-74, ref: 18-24	-1.17	-1.78 – -.55	<.001**
Education: high-school degree (ref: university degree)	.32	-.08 – .72	.116
Education: *Realschule* (ref: university degree)	-.13	-.73 – .46	.653
Education: *Hauptschule* (ref: university degree)	-.19	-1.35 – .96	.742
Education: other (ref: university degree)	.41	-.22 – 1.05	.199
Gender: male (ref: Female)	-.31	-1.01 – .39	.38

Note. Dependent Variable: adherent safety behavior (ASB). Total R^2=0.414, F(13, 124)= 6.936, p≤.001, n=138. ** p≤.01 * p≤.05

Table S2. Regression Coefficients Predicting DSB.

	Estimates	*CI*	*p*
Intercept	1.04	-1.13 – 3.21	.344
Diagnosis of CF	.76	.23 – 1.30	.005**
COVID-19-related fear	.22	.07 – .37	.004**
Subjective level of information	.03	-.28 – .33	.871
Age: 25-34, ref: 18-24	.19	-.54 – .91	.614
Age: 35-44, ref: 18-24	.22	-.57 – 1.02	.579
Age: 45-54, ref: 18-24	.14	-.74 – 1.02	.753
Age: 55-64, ref: 18-24	.39	-.78 – 1.57	.509
Age: 65-74, ref: 18-24	-.48	-1.98 – 1.03	.531
Education: high-school degree (ref: university degree)	.29	-.39 – .97	.394
Education: *Realschule* (ref: university degree)	.18	-.69 – 1.05	.681
Education: *Hauptschule* (ref: university degree)	-.38	-1.74 – .97	.574
Education: other (ref: university degree)	.7	-.36 – 1.76	.195
Gender: male (ref: Female)	.66	-.24 – 1.56	.148

Note. Dependent Variable: dysfunctional safety behavior (DSB). Total R^2=0.196, F(13, 124)=3.169, p≤.001, n=138. ** p≤.01 * p≤.05
